# White horses – non-coding sequences drive premature hair greying and predisposition to melanoma

**DOI:** 10.48101/ujms.v129.10626

**Published:** 2024-04-02

**Authors:** Leif Andersson

**Affiliations:** a Science for Life Laboratory, Department of Medical Biochemistry and Microbiology, Uppsala University, Uppsala, Sweden; b Department of Veterinary Integrative Biosciences, College of Veterinary Medicine and Biomedical Sciences, Texas A&M University, College Station, TX, USA

**Keywords:** horse, melanoma, pigmentation, enhancer, CNV, STX17, NR4A3

## Abstract

The *Grey* allele in horses is causing premature hair greying and susceptibility to melanoma. The causal mutation is a 4.6 kb tandem duplication in intron 6 of the *Syntaxin 17* gene. A recent study demonstrated that the most common allele at the *Grey* locus (*G3*) involves three tandem copies of this sequence, whilst a more rare allele (*G2*) has two tandem copies and the wild-type allele (*G1*) only one copy. The *G3* allele is causing fast greying and high incidence of skin melanoma, whereas the *G2* allele is causing slow greying and no obvious increase in melanoma incidence. Further somatic copy number expansion has been documented in melanoma tissue from Grey horses. Functional studies showed that this intronic sequence acts as a weak melanocyte-specific enhancer that becomes substantially stronger by the copy number expansion. The *Grey* mutation is associated with upregulated expression of both *Syntaxin 17* and the neighbouring *NR4A3* gene in Grey horse melanomas. It is still an open question which of these genes is most important for the phenotypic effects or if causality is due to the combined effect of upregulation of both genes. Interestingly, RNAseq data in the Human Protein Atlas give support for a possible role of *NR4A3* because it is particularly upregulated in human skin cancer, and it belongs to a cluster of genes associated with skin cancer and melanin biosynthesis. The *Grey* mutation and its association with melanoma provide a possibility to study the path to tumour development in numerous Grey horses carrying exactly the same predisposing mutation.

## Introduction

**Figure 1:**
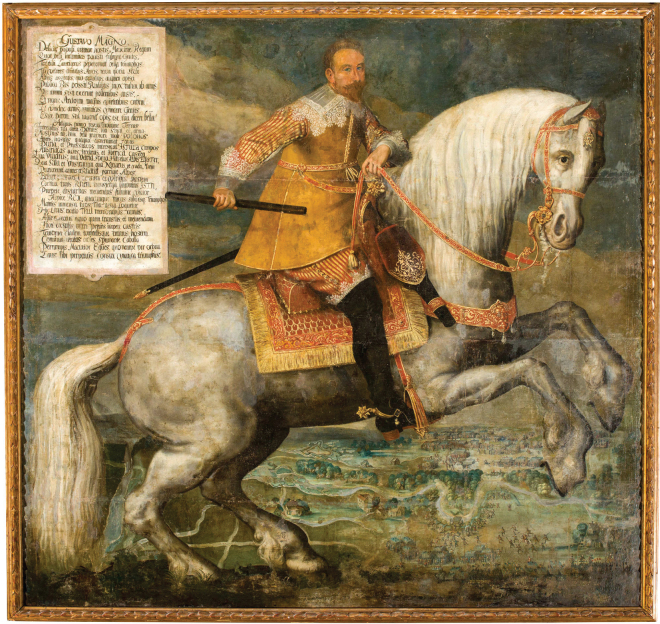
Swedish king Gustav II Adolf on a white horse with the city of Rain am Lech in the background. The battle at Rain took place on April 15, 1632 during the Thirty Year’s War. This painting from 1636 by Cornelius Arendtz is exhibited in the main hall (2^nd^ floor) of the University house at Uppsala University. Reproduced with permission from Uppsala University and Västmanlands-Dala nation, student nation in Uppsala.

Greying with age in horses is caused by an autosomal dominant mutation (1). These horses are born fully pigmented, but the greying process starts already during their first year of life, and by 6 years of age or later, the horses are usually shiny white. The greying process only affects hair pigmentation, whilst skin pigmentation is not affected or is even darker than that in non-grey horses. Furthermore, vision and hearing are not affected as is the case for instance for *MITF* (microphtalmia-transcription factor) mutations causing pigmentation phenotypes in humans and animals (2–4). Thus, a horse carrying the *Grey* mutation is as fit as any other horse. These horses compete successfully on the race track, which is a very demanding test of fitness. The fitness and beauty of white horses have made them extremely popular, and the phenotype occurs in many horse breeds. A famous example where white horses occur at a very high frequency is the Lipizzaner breed used by the Spanish riding school in Vienna. White colour in horses is, in fact, one of the most charismatic phenotypes in animals, and they have had a huge impact on human culture. One of the first written records of white horses is from the Greek historian Herodotus who describes that the Persian emperor Xerxes kept sacred white horses already about 2,500 years before present. White horses occur frequently in art from medieval time and onwards and have most certainly inspired the myths about for instance the Unicorn and Pegasus. Numerous kings, queens and other celebrities have been depicted riding a white horse (Figure 1). The *Grey* mutation arose most likely subsequent to domestication because in wild horses, there is selection for camouflage and most likely strong selection against white colour.

Greying with age involves three distinct phenotypes, and besides the dramatic effect on hair pigmentation it also predisposes to skin melanoma and vitiligo. It has been estimated that 50–80% of all Grey horses have skin melanomas when they are 15 years of age or older (5–7). These occur as dark nodules in glabrous skin, under the tail, at lips and on eye lids. These melanomas are jet-black and are usually benign but can cause discomfort to the horse, and a fraction of horses get wide-spread internal melanomas that becomes a lethal disease (6–8). The development of vitiligo is not well studied but is most likely an autoimmune disorder that develops subsequent to melanomas (1).

The aim of this mini-review is to summarise the current knowledge about the genetic basis for this fascinating phenotype, and why a copy number expansion of an intronic sequence causes both loss of hair pigmentation and melanoma.

## Copy number variation of a melanocyte-specific enhancer is the causal mutation

**Figure 2:**
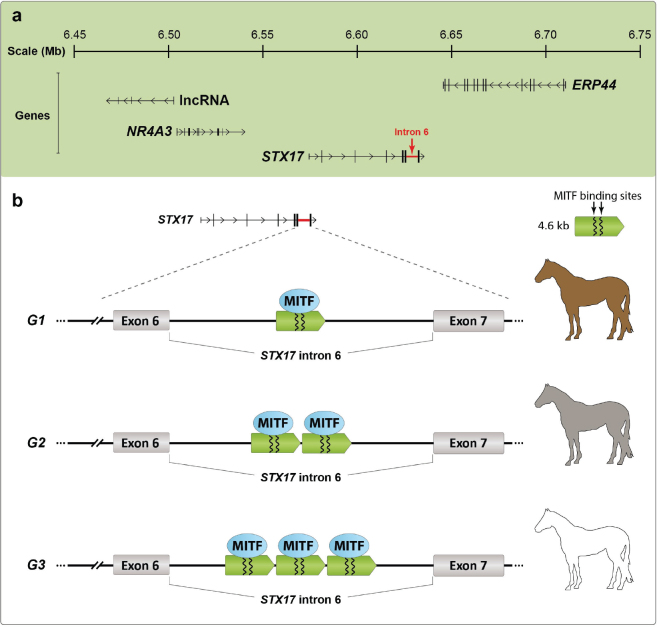
The 4.6 kb *STX17* duplicated sequence and its association with horse phenotypes. (a) The genomic region harbouring the *Grey* mutation. *STX17* and three of the flanking genes and the location of the *STX17* copy number expansion in intron 6 are indicated. (b) The constitution of alleles and their phenotype associations. The predicted interaction of the transcription factor MITF with two binding sites in each copy of the 4.6 kb sequence is schematically illustrated.

The *Grey* locus was mapped to horse chromosome 25 by classical linkage analysis (9). Since this rather broad genomic region did not contain any obvious candidate genes for hair pigmentation or skin melanoma, an Identical-by-Descent (IBD) approach for high resolution mapping was used under the assumption that the causal mutation has been inherited from a shared common ancestor in which the mutation originated. In a genetic screen comprising more than 700 Grey horses representing eight different breeds, a 350 kb IBD segment shared by all horses was identified (1). No exception to this rule has yet been reported, providing evidence that all Grey horses world-wide trace back to a single common ancestor in which the mutation arose. This is a classical selective sweep that has occurred during the evolution of domestic animals in which a mutation underlying a favoured phenotype quickly becomes widespread. The IBD region contains four genes: *NR4A3* (nuclear receptor subfamily 4 group A member 3), *STX17* (syntaxin 17), *ERP44* (endoplasmic reticulum protein 44) and *INVS* (inversin) (Figure 2a). None of these genes had previously been reported to be associated with variation in hair pigmentation or melanoma, and none of the genes carried any changes in coding sequences. However, further characterisation of the IBD region revealed a 4.6 kb duplication in intron 6 of *STX17* as the only unique sequence change present in all tested Grey horses (1).

A diagnostic test was developed for the presence of the intronic 4.6 kb duplication in *STX17*. This test was used to genotype almost 700 Grey Lipizzaner horses with extensive phenotypic records (1). This analysis demonstrated that *Grey* homozygotes compared with *Grey* heterozygotes grey faster, become more pure white and have a significantly higher incidence of melanoma and vitiligo, demonstrating a clear dosage effect for this mutation. However, *Grey* heterozygotes had a much higher-incidence of dark pigment spots. The latter is called flea-bitten grey and is a common phenotype in Grey horses, but almost all horses with this phenotype are heterozygotes. A possible explanation is that the *Grey* duplication may be inactivated epigenetically or that the extra copy is lost somatically. The likelihood that this occurs in the same cell on both chromosomes in a homozygous individual is small, compared with the likelihood that it occurs on the single *Grey* chromosome in cells of a heterozygous individual.

The identification of an intronic duplication in *STX17* as the causative mutation (1) resulted in an enigma. Why should this intronic mutation in a gene that at the time was poorly characterised and had never been associated with pigmentation or cancer biology has a spectacular effect on pigmentation and predispose to melanoma? Expression analysis of Grey horse melanoma tissue first demonstrated that both *STX17* and *NR4A3* show upregulated expression from the *Grey* chromosome in heterozygotes (1). Thus, the duplication constitutes a cis-acting regulatory mutation. A bioinformatic analysis revealed that the 4.6 kb sequence contains regions that show high sequence conservation amongst mammals, and the most well conserved part contains two binding sites for MITF (10); MITF is a master regulator of gene expression in pigment cells (11). A construct with the most highly conserved region fused with a minimal promoter, and Luciferase reporter was used for transfection experiments in mouse melanocyte (melan-a) and myoblast (C2C12) cell lines. This experiment demonstrated that the duplicated sequence has melanocyte-specific enhancer activity (10). This was further validated by generating transgenic zebrafish carrying transgenes with one or two copies of the 4.6 kb horse sequence, a minimal promoter and a green fluorescent protein (GFP) reporter. The zebrafish with a construct mimicking the *Grey* mutation carrying two copies of the horse sequence (STX17-DUP) but not those carrying the wild-type construct (STX17-1X) showed GFP expression in pigment cells (melanophores) (10). Furthermore, knock-down of MITF expression in transgenic zebrafish using morpholinos abolished GFP expression in STX17-DUP individuals. The results confirm that the duplicated sequence acts as a melanocyte-specific enhancer, and that the interaction with MITF is critical for enhancer activity.

## Copy number variation determines speed of greying and melanoma susceptibility

A recent study based on digital-PCR showed that most *Grey* chromosomes carry three tandem copies of the duplicated sequence rather than two copies (12). This finding suggested that there may exist further copy number variation of this sequence because it is well established that the presence of tandem copies of identical sequences tend to be unstable, and new alleles with another copy number may emerge due to unequal crossing-over or slippage during meiosis (13). We therefore decided to explore if copy number variation of the 4.6 kb *STX17* sequence may explain the dramatic difference in the speed of greying that is present in some breeds, for instance, in Connemara ponies. Copy number variation was determined using digital PCR, whole genome-sequencing and sequence capture followed by Nanopore sequencing (14). The analysis of a half-sib pedigree segregating for slow and fast greying demonstrated that slow-greying horses carry chromosomes with two tandem copies of the 4.6 kb *STX17* sequence, whereas fast greying horses carry a tandem triplication (14). Based on this result, a new nomenclature for the *Grey* locus has been established as follows: *G1* – one copy, wild type; *G2* – two copies, slow greying; *G3* – three copies, fast greying (Figure 2b). In contrast to *G1/G3* horses that show a high incidence of melanomas, no elevated risk compared with wild-type horses was noted for *G1/G2* horses (14). Thus, there is a remarkable dosage effect of the *Grey* mutation, increasing the copy number of the 4.6 kb *STX17* sequence shows a strong positive correlation with both speed of greying and incidence of melanoma (Table 1). It may be a threshold effect as regards the predisposition to melanoma, since none out of 25 *G1/G2* horses 15 years of age or older showed melanoma, in contrast to a >50% incidence amongst *G1/G3* heterozygotes of the same age (14).

**Table 1:** Relationship between genotype and phenotype at the *Grey* locus in horses.

Genotype	Speed of greying	Incidence of melanoma
*G1/G1*	non-grey	Low
*G1/G2*	slow	Low
G2/G2*	intermediate?	Low?
*G1/G3*	fast	high
G2/G3*	fast	high
*G3/G3*	very fast	very high

* Genotype–phenotype relationships are uncertain since the phenotype of only one *G2/G2* homozygote and one *G2/G3* heterozygote has been reported so far (14).

The strong correlation between germ-line dosage of the 4.6 kb *STX17* sequence and the incidence of melanoma in combination with the fact that tandem duplications and triplications show genome instability suggests that an additional somatic copy number expansion may occur in melanomas. This was in fact documented by quantitative PCR analysis of Grey horse melanoma samples and cell lines (15). A somatic copy number expansion of the 4.6 kb *STX17* sequence was noted in two out of four melanoma cell lines and in three out of four melanoma tissue samples. The result strongly suggests that an elevated copy number of the 4.6 kb *STX17* sequence is a driver mutation in these tumours.

## Why does the copy number expansion of the 4.6 kb ***STX17*** sequence predispose to melanomas in horses?

Expression analysis using grey horse melanomas showed that both *STX17* and the neighbouring gene *NR4A3* (Figure 2a) show upregulated expression from the *Grey* allele in heterozygous horses (1). Further characterisation of Grey horse melanomas and cell lines revealed constitutive activation of the ERK pathway (16), a characteristic feature shared with human melanomas. However, the exact mechanism leading to melanomagenesis is still poorly understood but both *STX17* and *NR4A3* have possible links to tumour development including relevance for melanomas. Syntaxin 17 is a SNARE protein with a critical function in the autophagy pathway (17), which has been proposed as a target for melanoma therapy in humans (18). *NR4A3* encodes an orphan nuclear receptor for which the function is only partially known but *NR4A3* fusion genes are associated with tumour development such as extraskeletal myxoid chondrosarcoma (19, 20). Interestingly, RNAseq data in the Human Protein Atlas (www.proteinatlas.org; accessed February 25, 2024) show that *NR4A3* RNA expression is particularly enriched in skin cancer, and that this gene belongs to a cluster of 199 genes associated with skin cancer and melanin biosynthesis. In contrast, *STX17* shows low cancer specificity in the same database. These data clearly strengthen upregulated *NR4A3* expression as a plausible candidate causal mechanism for melanoma susceptibility, which needs to be further explored. It is also worth noticing that the transcription of a long non-coding RNA (lncRNA) is initiated on the opposite strand just upstream of *NR4A3* both in horses (Figure 2a) and in human (http://genome.ucsc.edu; (GRCh38/hg38); accessed February 27, 2024). The function of this lncRNA is not well characterised but the very near vicinity of the start of transcription for *NR4A3* and this lncRNA suggests that it may contribute to the regulation of *NR4A3* expression, and if so, a regulatory mechanism that may be affected by the *Grey* mutation.

## Concluding remarks

The genetic evidence for that the copy number expansion of the 4.6 kb *STX17* sequence is the causal mutation both for hair greying and for predisposition to melanoma is now overwhelming. This is evident from 1) the presence of the duplication or triplication in thousands of tested grey horses world-wide, and there is no exception to this rule and 2) the remarkable correlation between the dosage of the 4.6 kb sequence and phenotype (Figure 2b; Table 1). Further somatic copy number expansion in tumours implies that the mutation not only predisposes to tumour development but also constitutes a driver mutation (15). However, the cellular mechanisms of how the duplication of this melanocyte-specific enhancer causes hair greying, and predisposes to melanoma are still poorly understood. This illustrates how difficult it is to establish the mechanism for how a non-coding mutation contributes to tumour development. Besides the horse data, there are no published data that give a clear indication why a copy number expansion of this evolutionary conserved sequence should predispose to melanoma. A PubMed search for ‘STX17+melanoma’ gives only one hit to a non-horse study, namely, a human study reporting that no association between *STX17* polymorphisms and cutaneous malignant melanoma was noted in a study comprising 1,560 cases and 1,650 controls (21). Further studies of this interesting mutation and its spectacular phenotypic effects are justified because it gives an opportunity to understand tumour development emanating from the same predisposing mutation. Further knowledge may eventually lead to an efficient treatment of a disorder that affects millions of horses world-wide.
